# Pathologic Remodeling of Endoneurial Tubules in Human Neuromas

**DOI:** 10.7759/cureus.2087

**Published:** 2018-01-18

**Authors:** Michael Karsy, Cheryl A Palmer, Mark A Mahan

**Affiliations:** 1 Department of Neurosurgery, University of Utah; 2 Department of Pathology, University of Utah

**Keywords:** endoneurial tubule, extracellular matrix, laminin, peripheral nerve injury, schwann cell

## Abstract

Background: Laminins are extracellular matrix proteins that participate in endoneurial tubule formation and are important in the regeneration of nerves after injury. They act as scaffolds to guide nerves to distal targets and play a key role in neurite outgrowth. Because there is evidence that laminin architecture affects nerve regeneration, we evaluated endoneurial tubules by examining the laminin structure in clinical samples from patients with nerve injuries.

Methods: In a retrospective review of eight nerve injury cases, we evaluated nerve histology in relation to clinical history and injury type. The immunohistochemical delineation of the laminin structure in relationship with the neuroma type was performed.

Results: Five cases of upper-trunk stretch injuries—four from childbirth injury and one from a motorcycle accident—and three cases of nerve laceration leading to neuroma formation were examined. In the upper-trunk stretch injuries, avulsed nerves demonstrated no neuroma formation with a linear laminin architecture and a regular Schwann cell arrangement, but increased fibrous tissue deposition. For neuromas-in-continuity after a stretch injury, laminin immunohistochemistry demonstrated a double-lumen laminin tubule, with encapsulation of the Schwann cells and axonal processes. Nerve laceration leading to stump neuroma formation had a similar double-lumen laminin tubule, but less severe fibrosis.

Conclusions: In nerve injuries with regenerative capacity, endoneurial tubules become pathologically disorganized. A double-lumen endoneurial tubule of unclear significance develops. The consistency of this pattern potentially suggests a reproducible pathophysiologic process. Further exploration of this pathophysiologic healing may provide insight into the failure of programmed peripheral nerve regeneration after injury.

## Introduction

Traumatic peripheral nerve injury (PNI) occurs in a wide array of situations, including laceration, concussion, stretch, rupture, or avulsion of peripheral nerves. PNI occurs in 2.8% of all trauma patients, with an incidence of 13–23:100,000 persons/year in developed countries, which represents a notably higher incidence than spinal cord injury [1-2]. Various mechanistic patterns of injury, levels of injury severity, and patient-specific factors (e.g., age, associated secondary injuries) can make PNI a heterogeneous disease process. A hallmark of severe injury is the development of a neuroma, which occurs with ineffective nerve fiber regeneration to its target tissue.

The term “neuroma” was first coined by Odier of Geneva in 1811 to describe deep lesions of nerves but did not distinguish nerve tumors from lesions with other mechanisms [3]. Current thought suggests that neuromas are the result of sprouting axons that exit from disrupted perineurium to form a fibrous, disorganized mass of fibroblasts and macrophages [4]. A related aspect, the neuroma-in-continuity, as described by Sunderland [5], describes a partial nerve injury, thickened tissue, Wallerian degeneration of fascicles, and the growth of nerve tissue out of endoneurial growth tubes but within the nerve epineurium. Extracellular matrix (ECM) scaffold proteins are postulated to play a role in nerve regeneration by organizing endoneurial tubules, macrophages, and Schwann cells [6].

We suspected that the pathophysiology of a neuroma-in-continuity is more than fibrous tissue that prevents neurite outgrowth because we recognize that there is fibrosis from surgical manipulation after every nerve grafting surgery. To evaluate our hypothesis that the endoneurial tubule may reveal aspects of the failure of regeneration, we assayed clinical samples from various neuroma-in-continuity and stump neuroma pathologic specimens. One of the important ECM proteins involved in the promotion of neurite outgrowth is laminin. Thus, we suspected that laminin may have the most to reveal about neuromas.

## Materials and methods

After receiving institutional review board approval with a waiver of informed consent, we performed a retrospective chart and pathological review to identify patients of the senior author (MM) who underwent surgical neurolysis and resection of neuromas as part of surgical repair. The pathological review was performed to ensure tissue was available. Histology was performed at the discretion of the senior author and neuropathologist (CP); it included hematoxylin and eosin (H&E), trichrome, and laminin immunohistochemistry (IHC) stains. Five-micrometer, formalin-fixed, paraffin-embedded sections of neuroma resections from all patients were cut at regular intervals and mounted on glass sides. H&E and trichrome stains were prepared on all specimens except for Cases Two and Four. Immunohistochemical staining was performed using laminin antibodies (Leica Biosystems, Wetzlar‎, Germany) at a dilution of 1:100. Staining was performed using the avidin-biotinylated peroxidase complex (ABC) method on a Ventana Staining system (Ventana Medical Systems, Inc., Arizona, United States) and counterstained with hematoxylin.

The means (ranges) of patient ages and follow-up, along with the mechanism of injury and descriptive histopathological findings, were analyzed. Statistical analysis was not performed with this limited sample.

## Results

The summary of all eight patients is shown in Table 1. Case descriptions are presented in the Supplemental Material. Five patients, four infants, and one young adult, with a mean age of 5.8±12.1 years (95% confidence interval (CI) 0.3, 27.4 years; median 0.5 years) had stretch-related/avulsion injuries; and three patients with a mean age of 40.1±14.4 years (CI 26.9, 55.5 years) had transection injuries. Stretch-related injuries occurred in two males and three females, mostly infants. Transection injuries occurred in two males and one female, all adults. At surgery, there were five neuroma-in-continuity injuries, four from birth injuries and one stab wound; two stump neuromas, both iatrogenic; and one spinal nerve avulsion injury, with discontinuity of the peripheral nerve from the spinal cord.

**Table 1:** Summary of peripheral nerve injury cases and staining patterns

Case	Age (yr)	Sex	Mechanism	Injury pattern classification	Neuroma pattern	Staining		
						H&E	Trichrome	Laminin
1	0.5	M	Birth brachial plexopathy	Stretch-related	Neuroma-in-continuity	Whorl-like perineurium	Fibrotic interneuron areas	Aberrant double-lumen
2	0.5	F	Birth brachial plexopathy	Stretch-related	Neuroma-in-continuity	Whorl-like perineurium		Aberrant double-lumen
3	0.3	F	Birth brachial plexopathy	Stretch-related	Neuroma-in-continuity	Whorl-like perineurium	Fibrotic interneuron areas	Aberrant double-lumen
4	0.5	F	Birth brachial plexopathy	Stretch-related	Neuroma-in-continuity	Whorl-like perineurium		Aberrant double-lumen
5	27.4	M	Trauma to brachial plexus from motorcycle	Avulsion	No neuroma	Normal pattern, wavy	Fibrotic perineurial scar	Wavy linear fibers
6	26.9	F	Orthopedic resection of common peroneal nerve	Transection	Stump neuroma	Whorl-like perineurium	Fibrotic perineurial scar	Aberrant double-lumen
7	37.9	M	Iatrogenic femoral nerve injury from inguinal herniorrhaphy	Transection	Stump neuroma	Granular-like perineurium	Fibrotic perineurial scar	Aberrant double-lumen
8	55.5	M	Work-related median nerve laceration	Transection	Neuroma-in-continuity	Granular-like perineurium	Fibrotic perineurial scar	Aberrant double-lumen

The four infants with stretch-related injuries demonstrated classic neuroma formation on histology, as did the three patients with laceration/iatrogenic injury. The single patient with the nerve root avulsion injury did not demonstrate neuroma formation.

Stretch neuroma-in-continuity

**Figure 1:**
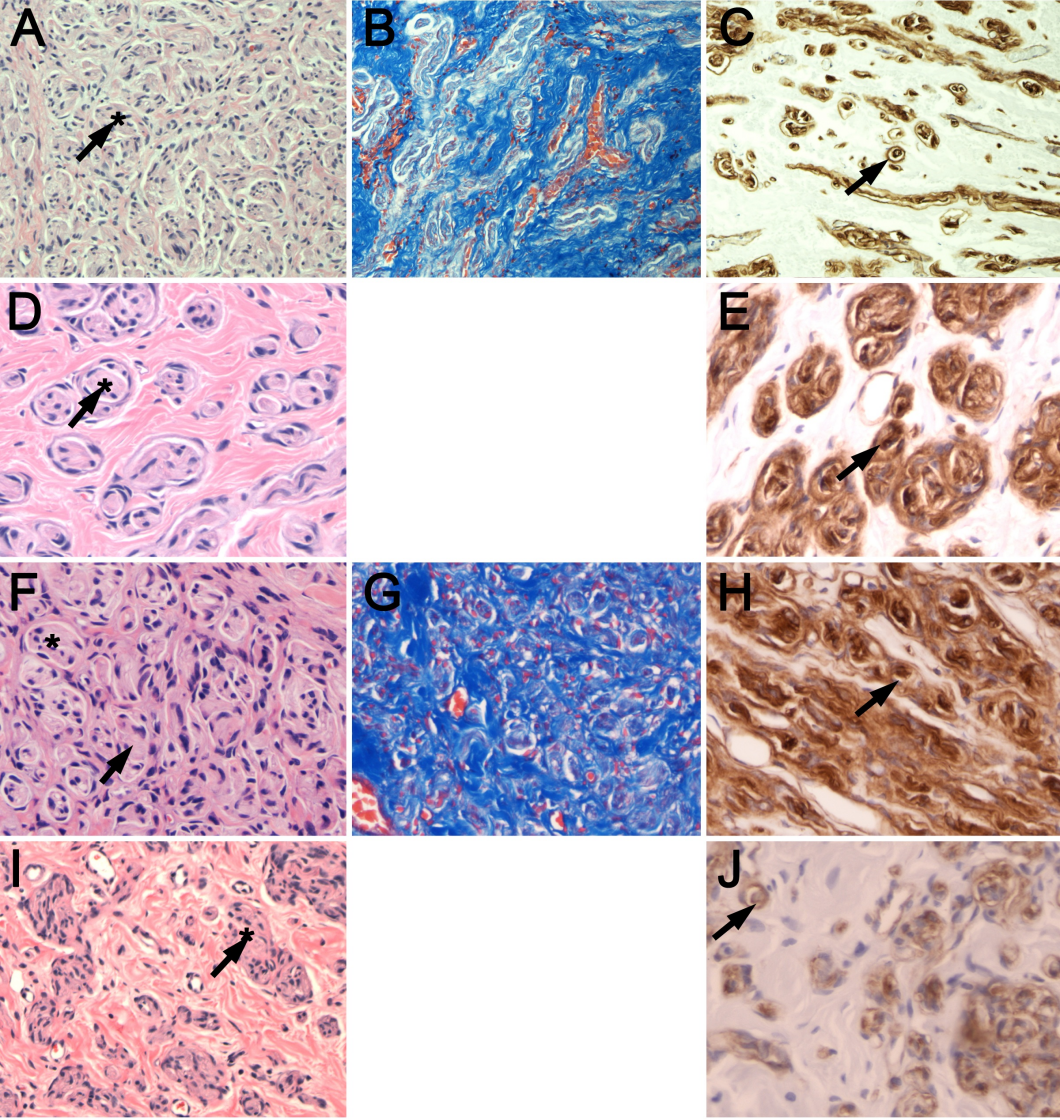
Histology of pediatric brachial plexus stretch neuromas-in-continuity (A, D, F, I) H&E, (B, G) trichrome, and (C, E, H, J) laminin staining of resected neuromas-in-continuity from infants with birth stretch-related injury is shown. Monotonous cellularity and whorl-like endoneurium (asterisk) and extensive intraneural fibrotic scar are seen on H&E and trichrome stains. Laminin staining showing uneven, abnormal double-lumen endoneurial tubules (arrows) reflective of severely aberrant regeneration. Case One = A, B, C, ×100); Case Two = D, E, ×200; Case Three = F, G, H, ×200; Case Four = I, J, ×100.

On H&E stains, neuromas from infants with stretch-related injuries showed a monotonous cellularity and whorl-like perineurium along extensive interfascicular fibrotic scars (Figure 1, A, D, F, I). Trichrome stain showed perineurial fibrotic scar and disorganized nonlaminar architecture (Figure 1, B, G). Laminin IHC showed aberrant ECM formation, with uneven and abnormal double-lumen endoneurial tubules (Figure 1, C, E, H, J).

Stretch avulsion injury

**Figure 2:**
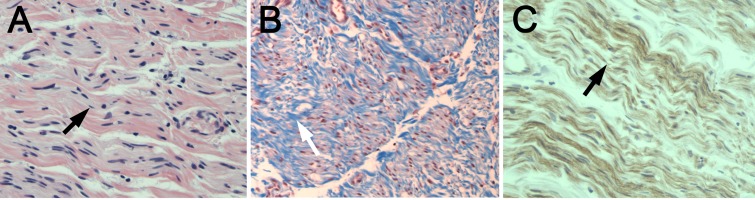
Histology of brachial plexus avulsion injury (A) H&E, (B) trichrome, and (C) laminin staining (all ×200) of the proximal end of the lower trunk from an adult with avulsion is shown (Case Five). The lower trunk was trimmed prior to transfer to the C7 spinal nerve. Parallel, wavy fibers (black arrow, A) with minimal fibrotic scar (white arrow, B) and laminar laminin (black arrow, C) with minimal disruption is seen. Minimal regeneration is seen along with limited fibrosis.

Nerve tissue from the trunks of a brachial plexus after avulsion from the spinal cord showed parallel, wavy fibers with minimal fibrotic scar on H&E (Figure 2, A-C). Nerve fibers showed evidence of stretch, with relative straightening of the nerve fibers and wide fiber spacing. Laminin stain reflected the H&E showing laminar, parallel extracellular protein deposition with minimal disruption (Figure 2A).

Laceration neuromas

**Figure 3:**
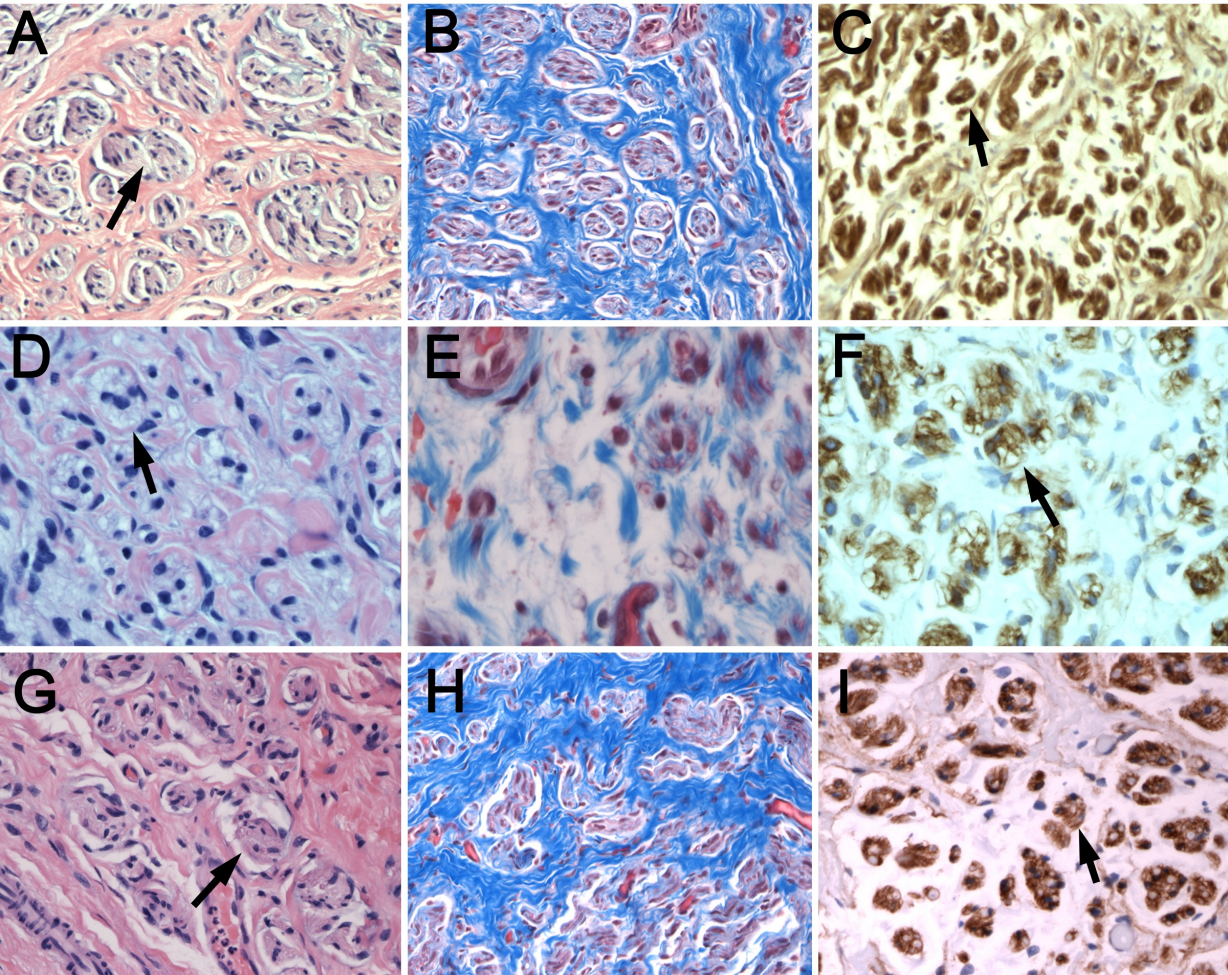
Histology of nerve laceration injuries (A, D, G) H&E, (B, E, H) trichrome, and (C, F, I) laminin staining of neuromas from adult patients with transections is shown. Well-encapsulated endoneurial tissue, heterogeneous nuclei, with reduced interneural fibrotic scars (black arrows, A, D, G) as compared with samples from neonatal stretch injuries. Double-lumen endoneurial tubules are seen (black arrows, C, F, I) are similar to those from birth stretch injuries. Case Six = A, B, C, ×200; Case Seven = D, E, F, ×400; Case Eight = G, H, I, ×200.

Neuromas from the transection injuries were similar in microscopic appearance to the stretch-related injuries. H&E stains showed well-encapsulated perineurial tissue, heterogeneous nuclei, with reduced interneural fibrotic scars (Figure 3, A, D, G). Trichrome reflected the presence of fibrotic intraneural tissue (Figure 3, B, E, H). Laminin IHC showed separated groupings of double-lumen endoneurial tubules in all three cases (Figure 3, C, F, I).

## Discussion

The ECM, Schwann cells, and signal transduction from trophic factors play an important role in axon regeneration and functional recovery [7]. These three factors have been suggested to work in concert in promoting successful regeneration; however, the tissue architecture mediating this process is not clearly understood. Upon losing contact with an axon because of Wallerian degeneration, Schwann cells revert to an immature, proregenerative state [8]. When transformed to this state, they proliferate and signal macrophage infiltration to clear debris. Schwann cells reorganize into columns along the ECM, termed the Bands of Büngner, to guide neuronal axon growth [9].

In the case of a neuroma-in-continuity, a distinct pathophysiology likely occurs to prevent the choreography of neurite, Schwann cell, and ECM from successful regeneration. We hypothesized that the endoneurial tubule may play a role in the failure of regeneration, and we selected laminin as a potential marker of the particular failure within the endoneurial tubule.

Laminins play an important role in axonal guidance. Laminin has been used as a substrate for nerve conduits to guide axonal growth in a variety of settings [10-11] and has been used during tissue engineering approaches [12-13]. Laminin specifically induces signaling pathways, including PI-3-kinase [14]. Within Schwann cells, an early pro-myelinating pathway is driven by neuregulin 1 while a later anti-myelinating pathway is driven by laminin [14]. Schwann cells lacking laminin induction were shown to express decreased schwannomin (ser518) phosphorylation, as well as CDC42 and Rac1 activation [15]. Furthermore, decreased levels of these proteins reduced Schwann cell-dependent myelination. Laminins were able to enhance the phosphorylation of IκB and p65 NF-κB signaling proteins in schwannoma cells [16]. A study by Chen and Strickland [17] demonstrated the importance of laminins using a Cre-loxP system to disrupt laminin γ1 in Schwann cells. This model showed the motor deficits of laminin resulted in hind leg paralysis, tremor, and Schwann cell inability to differentiate and synthesize myelin proteins as well as Schwann cell apoptosis. In addition, after a sciatic nerve crush, axons showed significantly impaired regeneration. These results suggest that laminin plays a key role in organizing neurite outgrowth as well as proper signaling to regulate regeneration.

**Figure 4:**
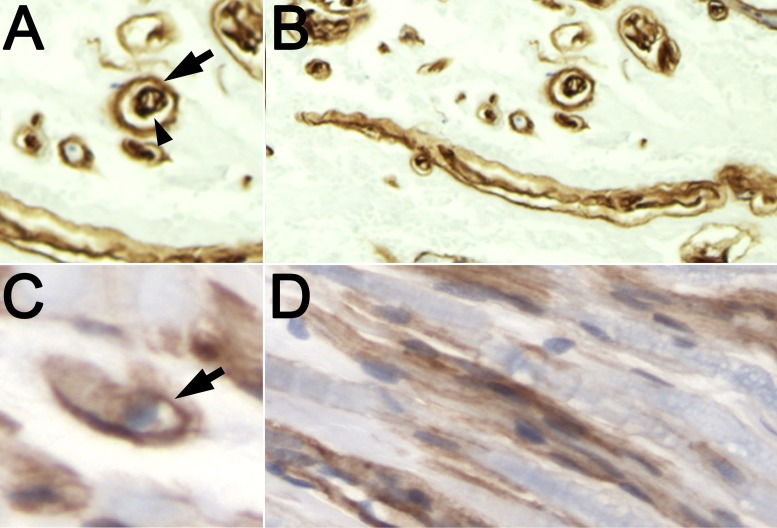
Double-lumen laminin pattern compared with typical laminin pattern (anti-laminin IHC and hematoxylin) (A,B) neuroma-in-continuity specimen presented with cross-sectional (A) and longitudinal (B) slicing of the endoneurial tubule. The outer lumen (arrowhead) typically has thinner staining and an eccentric nucleus outside the laminin. The inner lumen (arrow) laminin is irregular, appears to have multiple channels, and may contain one or more nuclei. (C,D) normal nerve specimen removed for non-pathologic indications presented with cross-sectional (C) and longitudinal (D) slicing of the endoneurial tubule. Laminin staining is around a single channel, no more than one internal nucleus (C) and is thin around Schwann cells (D) (all ×400). IHC: immunohistochemistry

Our pathologic specimens demonstrated a consistent double lumen of a laminin-encasing axon and Schwann cells (Figure 4) in all forms of neuromas. The origins and consequences of this histopathologic observation are unknown. One possible cause may be the pathophysiologic remodeling of damaged ECM by regenerating axons and Schwann cells. Alternatively, the invasion of inflammatory cells after trauma may also participate in the pathology of extracellular proteins.

Two interesting features are notable. First, the pattern of a double-lumen tubule was consistent, regardless of whether the lesion was a neuroma-in-continuity or a stump neuroma and of the age of the patient, suggesting a conserved or consistent response to severe injury. Second, injured or regenerating axons seem to be required. As shown in the histology of the avulsed spinal nerves, where motor neurons are not present and the distal sensory axons are presumably intact, the architecture of the laminin tubules was unremarkable. Because Schwann cells produce the ECM and regenerating axons appear to be essential for the formation of the double-lumen tubule, it would seem to be a shared process. It is possible that loss of endoneurial tubule integrity produces axon–Schwann cell-mediated remodeling, as has been shown when large somatic fibers remodel the smaller endoneurial tubules of autonomic nerves [18-19]. However, much further work is necessary to identify the mechanism of this laminin deposition.

There are several limitations of this study. The sample is currently a small sample of overall nerve injury patterns. There was some heterogeneity of IHC staining among samples. In addition, we have only one sample from an avulsion injury, which served as a key comparison.

## Conclusions

Our study used laminin antibodies to assess pathophysiologic regeneration in neuromas. Laminin IHC showed disorganized double-lumen endoneurial tubules in pathologic specimens of neuroma-in-continuity and stump neuromas, whereas avulsion injury maintained good nerve architecture and a relatively normal laminin pattern. This observation of the pathologic remodeling of endoneurial tubules during neuroma formation suggests pair interaction between regenerating axons and Schwann cells in response to injury to the ECM. Better understanding these patterns may help to generate better-directed treatment approaches.

## Appendices

Case descriptions

Case One

A 6.5-month-old boy with shoulder dystocia during vaginal delivery due to macrosomia and anoxic injury at time of delivery presented with a flaccid left arm and progressive waiter’s tip position and active grasp (Figure 1 A–C). Magnetic resonance imaging (MRI) demonstrated absent ventral rootlets at C8 with a clumping of nerve roots and hyperintensity of the C5–C7 roots suggestive of a neuroma. The patient underwent the grafting of C5 to the suprascapular nerve, C5 to the axillary nerve, and C6 to the lateral cord.

Case Two

A six-month-old girl presented with shoulder dystocia and flail right arm after birth by vaginal delivery (Figure 1 D, E). MRI demonstrated neuromas-in-continuity of the upper trunk along with a pseudomeningocele at C7. She underwent a brachial plexus neurolysis and a cabled graft repair of C5 to the posterior division of the upper trunk and the supraclavicular nerve and of C6 to the anterior division of the upper trunk.

Case Three

A four-month-old girl presented with a flail left arm after shoulder dystocia complicated by macrosomia and prolonged labor during birth by vaginal delivery (Figure 1 F–H). Examination at the time of birth showed an inability to move the left arm with a waiter’s tip position. MRI showed traction injury to C5-7 with neuroma formation. The patient underwent a brachial plexus neurolysis and a cabled graft repair of C5 to the posterior division and the suprascapular nerve and of C6 to the anterior division.

Case Four

A five-month-old girl presented with obstetrical brachial plexus injury secondary to macrosomia and prolonged descent (Figure 1  I, J). The neurological examination at presentation showed a flail arm in the waiter’s tip position without functional recovery. MRI demonstrated pseudomeningoceles at C6, C7, and T1. The patient underwent brachial plexus neurolysis and cabled graft repair of C5 to the posterior division and suprascapular nerve, C6 to the anterior division, and contralateral C7 to the lower trunk.

Case Five

A 27-year-old man presented four months after a motorcycle accident with associated clavicular fracture and pan-brachial plexopathy (Figure 2 A–C). MRI demonstrated absent rootlets at C8-T1 and a clumping of rootlets at C7, with an electromyogram (EMG) notable for modest denervation–reinnervation of the rhomboids. The patient underwent a multistage nerve transfer, including neurolysis of the brachial plexus, distal spinal accessory–to-suprascapular transfer, C6 transfer to the posterior division of the upper trunk, and ipsilateral C7 transfer to the lower trunk. A second-stage procedure performed two weeks later included transfers of the motor intercostal nerves to musculocutaneous and biceps branches and to a sural nerve graft for later, staged, free functioning muscle transfer, and transfers of sensory intercostal nerves to lateral cord contribution to the median nerve, lateral antebrachial cutaneous nerve, and medial antebrachial cutaneous nerve.

Case Six

A 26-year-old woman presented with significant left lateral leg pain after being struck in the fibular head with a softball 11 years prior (Figure 3 A–C). She had undergone over 29 knee surgeries for pain, including decompression of the common peroneal nerve, and underwent a common peroneal nerve resection with Bridle procedure (posterior tibialis tendon to the insertion of tibialis anterior) at another institution. MRI revealed a large neuroma at the common peroneal nerve resection. She underwent resection of the neuroma and transfer of the common peroneal nerve to the nerve pedicle of the short head of the biceps femoris muscle.

Case Seven

A 38-year-old man presented with continued left leg pain and new leg weakness three months after a revision herniorrhaphy with an intended division of the ilioinguinal and iliohypogastric nerves for pain control at an outside institution (Figure 3 D–F). He had a quadriceps muscle weakness of 1/5 on the Medical Research Council scale and diminished sensation in the femoral and saphenous cutaneous distributions. A pelvis MRI showed hyperintensity along the femoral nerves with a thickening of the femoral nerve suggestive of an iatrogenic neuroma. The patient underwent repair of the lacerated medial femoral nerve branches and definitive neurectomy of the genitofemoral and ilioinguinal nerves.

Case Eight

A 55-year-old man presented five months after accidentally stabbing his left medial forearm, six inches proximal to the distal crease, in a cattle-handling accident (Figure 3 G–I). His symptoms include persistent numbness in the 4^th^ and 5^th^ digits and atrophy of the distal ulnar distribution in the hand. EMG results show no sensory recording of the 5^th^ digit, prolonged latency and decreased conduction velocity of the ulnar motor nerve, and fibrillation with positive sharp waves of the first dorsal interosseous and abductor quinti minimi. MRI suggested partial ulnar nerve laceration. The patient underwent an ulnar nerve internal neurolysis and fascicular repair.
